# Visual Temporal Contrast Sensitivity in the Behaving Mouse Shares Fundamental Properties with Human Psychophysics

**DOI:** 10.1523/ENEURO.0181-18.2018

**Published:** 2018-08-29

**Authors:** Yumiko Umino, Rose Pasquale, Eduardo Solessio

**Affiliations:** Department of Ophthalmology, Center for Vision Research, SUNY Upstate Medical University, Syracuse, New York 13210

**Keywords:** forced-choice design, mouse, operant behavior, temporal contrast sensitivity, theory of signal detection

## Abstract

The mammalian visual system has a remarkable capacity to detect differences in contrast across time, which is known as temporal contrast sensitivity (TCS). Details of the underlying neural mechanisms are rapidly emerging as a result of a series of elegant electrophysiological studies performed largely with the mouse as an experimental model. However, rigorous psychophysical methods are necessary to pair the electrophysiology with temporal visual behavior in mouse. The optomotor response is frequently used as a proxy for retinal temporal processing in rodents. However, subcortical reflexive pathways drive the optomotor response rather than cortical decision-making areas. To address this problem, we have developed an operant behavior assay that measures TCS in behaving mice. Mice were trained to perform a forced-choice visual task and were tested daily on their ability to distinguish flickering from nonflickering overhead lights. Correct responses (Hit and Correct Rejections) were rewarded. Contrast, temporal frequency, and mean illumination of the flicker were the independent variables. We validated and applied the theory of signal detection to estimate the discriminability factor (*d*´), a measure of performance that is independent of response bias and motivation. The empirical contrast threshold was defined as the contrast necessary to elicit *d*´ = 1 and TCS as the inverse of the contrast threshold. With this approach, we established in the mouse a model of human vision that shares fundamental properties of human temporal psychophysics such as Weber adaptation in response to low temporal frequency flicker and illumination-dependent increases in critical flicker frequency as predicted by the Ferry–Porter law.

## Significance Statement

Rigorous psychophysical methods are necessary to pair the power of mouse genetics and electrophysiological studies with temporal visual behavior using the mouse as an experimental model. To address this problem, we have developed an operant behavior assay that measures temporal contrast sensitivity (TCS) in mice. Our assay is based on a forced-choice alternative response protocol and applies the theory of signal detection to generate an unbiased measure of sensitivity. With this approach, we measured TCS and established in the mouse a model of human vision that shares fundamental properties of human temporal psychophysics. This finding fills in a major gap in our knowledge that is of general interest to the visual science community studying retinal and central visual processing using the mouse as an experimental model.

## Introduction

Over much of the visual range, the visual system remains exquisitely sensitive to contrast (small differences in luminance; Shapley and Enroth-Cugell, 1984). Visual responses adaptively speed up as light levels increase, allowing humans to detect flicker at higher temporal frequencies than they can under dim ambient conditions (de Lange, 1958; Kelly, 1961). Although it has long been established that the mechanisms of contrast sensitivity and light adaptation reside largely in the retina (Dowling, 1967; Donner et al., 1990; Purpura et al., 1990; Tranchina et al., 1991; Seiple et al., 1992; Dunn et al., 2007; Smith et al., 2008), we are only now beginning to identify and understand their cellular and synaptic basis (Dunn et al., 2006, 2007; Jarsky et al., 2011; Oesch and Diamond, 2011; Ke et al., 2014; Sinha et al., 2017). The majority of these more recent studies were performed with the mouse in view of its practical advantages as an experimental model (Nishina and Naggert, 2003). A downside to the use of mouse is that temporal visual behavior in the mouse is poorly understood, making it difficult to establish the link between behavior and the electrophysiological output and/or its relevance to human vision (Parker and Newsome, 1998).

The optomotor reflex in mice is frequently used as a proxy for retinal temporal processing in rodents (Prusky et al., 2004; Umino et al., 2008, 2012). However, subcortical reflexive pathways drive the optomotor response rather than cortical and decision-making areas (Simpson, 1984; Douglas et al., 2005; Sun et al., 2006). In addition, the use of the optomotor response assay is suboptimal for the study of temporal contrast sensitivity (TCS) because (1) of the potential for interactions in the processing of speed versus temporal frequency properties of the drifting grating stimulus, and (2) the dynamic response range is substantially less than the retinal responses.

To address these problems, we have developed an operant conditioning assay that allows the determination of TCS in behaving mice. Although the temporal resolution of the optomotor response is limited to ∼12 Hz, and the flicker ERG responses extend to 24 Hz, we show that behavioral responses measured with our new operant approach extend to ∼40 Hz. Operant conditioning methods have previously been applied to test mouse vision, including absolute and spectral sensitivities (Nathan et al., 2006; Jacobs et al., 2007; Naarendorp et al., 2010) and spatial contrast sensitivity (Busse et al., 2011; Histed et al., 2012). One earlier behavioral study—using a water maze—reported temporal resolution (Nathan et al., 2006), while a more recent behavioral study—using a go/no-go approach—reported temporal contrast sensitivity in mice (Peinado Allina et al., 2017). Our operant behavior assay is based on a variation of the forced-choice alternative response protocol described by Jacobs et al. (2004) using a flicker discrimination technique and applies the theory of signal detection (TSD) to determine the discriminability factor (*d*´), an unbiased measure of performance. To validate the application of TSD to our mouse operant behavior, we confirmed that the receiver operating characteristic (ROC) curves implied by *d*´ can predict empirical ROC curves generated by extrinsically manipulating response bias (Macmillan and Creelman, 2005). With this approach, we measured the TCS and established in the mouse a model of human vision that shares fundamental properties of human temporal psychophysics, such as Weber adaptation, in response to low-temporal frequency flicker and illumination-dependent increases in critical flicker frequency (CFF), as predicted by the Ferry–Porter law (for review, see Shapley and Enroth-Cugell, 1984; Watson, 1986). This finding fills in a major gap in our knowledge that is of general interest and significance to the visual science community studying both retinal and central visual processing with the mouse as an experimental model. Given the novel nature of our behavioral approach, we provide a detailed description of the experimental setup, the animal training paradigms, and the validation of TSD as model of decision-making in the mouse.

## Materials and Methods

### Mouse genotype and husbandry: animal strains

We used C57BL/6J mice and “black” GNAT2^*cpfl3*^ mice (Chang et al., 2006) bred for more than seven generations into the C57BL/6J background. GNAT2^*cpfl3*^ mice carry a spontaneous point mutation in the GNAT2 gene that reduces cone phototransduction efficacy (Chang et al., 2006). C57BL/6J mice and black GNAT2^*cpfl3*^ are referred to as wild type (WT) and G2 in the text. Animals of either sex, 3–6 months of age, were tested in operant behavioral assays. Mice were maintained on a 14 h/10 h light/dark cycle, dark adapted for 1 h before experiments, and tested during the subjective day. All animal procedures in this study were approved by SUNY Upstate Medical University Institutional Animal Care and Use Committee (IACUC approval #297) and were conducted in accordance with the *Guide for the Care and Use of Laboratory Animals* (National Academy of Sciences) and in compliance with The Association for Research in Vision and Ophthalmology Statement for the Use of Animals in Ophthalmic and Vision Research. All efforts were made to minimize animal suffering.

### Operant conditioning assay to determine TCS in mice

**Figure 1.**
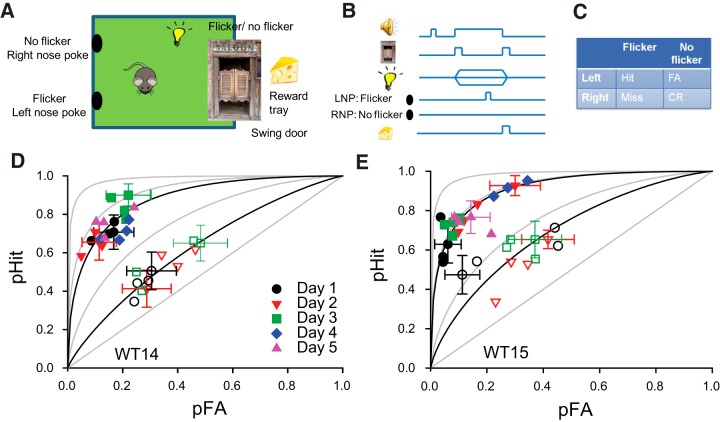
An operant behavior assay to determine temporal contrast sensitivity in the mouse. *A*, Layout of the experimental chamber used to measure TCS in mice with an operant behavior assay. *B*, Testing paradigm (for details, see Materials and Method). *C*, Performance scoring table. *D*, *E,* Probability of Hit versus FA for two different mice (WT14 and WT15) measured over the course of 5 successive days in response to 50% (open symbols) and 90% (closed symbols) contrast flicker and mean background illumination producing ∼8200 ph/s/μm^2^ at the retina (Table 1, light calibration details). Each symbol represents the Hit vs FA pair for 100 trial blocks measured during a daily session. A total of four blocks was measured in each session. Error bars indicate representative 95% intervals (estimated as per Macmillan and Creelman, 2005). In gray are ROC curves for *d*´ = 0, 1, 2, and 3, respectively. In black are ROC curves for *d*´ = 0.45 and 1.68 in ***D*** and *d*´ = 0.75 and 1.94 in ***E*** fitting Hit vs FA pairs for 50% and 90% contrast, respectively.

In our operant studies, mice are trained to detect and respond to a visual stimulus, an action that requires cortical input and decision-making. Here we describe this new approach. Operant tests are performed using a control and conditioning system (Lafayette Instrument) consisting of eight modular test chambers independently controlled by ABET II software (Lafayette Instrument). The user-defined program schedules the tests, records the responses, and presents reinforcement. Each chamber is equipped with a custom-built programmable LED-based light stimulus (505 nm central emission) placed overhead, a reward tray, and two nose-poke ports installed on the wall opposite to the reward tray (Fig. 1*A*). Placing the nose-poke ports in the wall opposite to the reward tray requires that the mice walk a sufficiently long path to the nose-poke port, thereby providing time for the development of the flicker stimulus before selection of the nose-poke port.

Each mouse is maintained on a food-restriction schedule sufficient to provide necessary motivation to learn and perform the behavioral task (food is restricted to achieve 80–85% of expected body weight). Mice are trained to perform the following forced-choice visual task: discriminate the presence or absence of flicker in the test light (food restriction details and training routine details are provided below). As indicated schematically in Figure 1*B*, a brief (20 ms) high-frequency tone is presented to alert the mice that they can initiate a trial. Trials are initiated when the mouse visits the reward tray. Following trial initiation, the light stimulus is presented concurrent with a cueing tone, both of which terminate when the animal responds or, in the absence of a response, after 12 s. A response is computed as a visit to the reward tray following a visit to the left or right nose-poke ports. Correct responses [which include both Hits and correct rejections (CRs); Fig. 1*C* and related text] are rewarded with a small amount of commercially available Ensure Nutrition Drink (∼5–10 μl; Abbott Laboratories) on a schedule that rewards 100% of the hits and 100% of the correct rejections. It routinely requires 20–25 d of training for mice to associate a particular response port in the wall of the behavioral chamber (left or right) with one of two possible stimulus alternatives (flicker or nonflicker).

Following training (see below for details), we test daily the ability of mice to detect flickering from nonflickering lights. Each session consists of 400 trials following a “warm-up” session of 300 corrective trials; 400 trials/session produces consistent day-to-day measures of performance. The duration of each session is ∼2–3 h. During the sessions, mice are continuously exposed to a fixed adapting light; the flicker test is a sinusoidally varying full-field illumination superimposed on the steady adapting light. The level of the nonflicker test light is matched to the adapting light. Stimulus variables defined by the experimenter are mean light intensity (*I*_o_), temporal frequency (*f_t_*) and Rayleigh (or Michelson) contrast (*C*). Stimulus onset is ramped over 1 s to reduce the generation of “spillover” frequencies that are generated by a step onset. For the estimation of *d*´ and the criterion location (CL), we applied the following relations (Macmillan and Creelman, 2005):

(1) $$d^{\prime }=z\left(\text{Hit}\right)-z\left(\text{FA}\right)$$

and

(2) CL=−0.5[z(Hit)+z(FA)],

where z(⋅) represents the *z* score of the probability of hits (Hit) or false alarms (FA) in a trial.

To derive an expression that relates CL to the reward schedule, we examined the relationship between the likelihood ratio β and the payoffs for each outcome (Macmillan and Creelman, 2005), as follows:

(3) $$\beta =e^{d^{\prime }CL}=\left\{\frac{R\left(\text{CR}\right)-R\left(\text{FA}\right)}{R\left(\text{Hit}\right)-R\left(\text{Miss}\right)}\right\}\frac{p\left(\text{flicker}\right)}{p\left(\text{nonflicker}\right)},$$

where *R*(CR), *R*(Miss), *R*(Hits), and *R*(FA) represent the payoffs for each outcome and *p*(flicker) and *p*(nonflicker) are the probability of flicker and nonflicker stimuli presentations. In our experimental conditions, *R*(Miss) = *R*(FA) *= 0*, and *p*(flicker) *= p*(nonflicker) = 0.5. Hence, from Equation 3 we derive the following:

(4) $$\text{CL}=-\frac{1}{d^{\prime }}\text{ln}\left[\frac{R\left(\text{Hit}\right)}{R\left(\text{CR}\right)}\right].$$

To account for the intrinsic bias and the slope of the relationship, we rewrite Equation 4 in the following terms:

(5) $$\text{CL}=\text{CL}\left(100\text{\%},100\text{\%}\right)-\frac{q}{d^{\prime }}\text{ln}\left[\frac{R\left(\text{Hit}\right)}{R\left(\text{CR}\right)}\right],$$

where *R*(Hit)/*R*(CR) is the schedule ratio and the constant term CL(100%,100%) accounts for the intrinsic bias exhibited by each mouse when tested with a balanced reward schedule (R(Hit) = R(CR) = 100%) and *q* is a free variable.

### Food restriction schedule

Mice were placed under food restriction (reduced amounts of food available *ad libitum*) before and during the testing period to maintain their body weight at 80–85% of their free feeding weight (adjusted in accordance with their growth curve). The protocol was used with adult mice, starting at 9–11 weeks of age. Animals placed in individual cages were given 10 calories/d dry food until their weight stabilized. We fed the animals with dry, grain-based dustless precision pellets (Bio-Serve Custom Diets) served as 500 and 45 mg pellets (catalog #F0171 and #F0165, respectively). We continued the process dropping in 1 calorie decrements until the weight of the animal reached the targeted 80% weight. At the start of the third week, we gave the mice 2 cc of vanilla Ensure in a cup in their cages daily. Liquid Ensure is an off-the-shelf balanced nutritional supplement that has proven to be an effective reward for mice performing in olfactory behavioral tasks (Youngentob and Margolis, 1999). The amount of dry food was adjusted accordingly to balance caloric input. Using this protocol, it takes 3–4 weeks to bring an animal to weight. We also increase gradually the relative amount of Ensure in the diet, such that by the end of this period, mice consume 3–4 ml of Ensure daily. To maintain caloric balance, the daily number of calories provided in the diet was adjusted depending on the number of calories consumed in the form of reward during test sessions. In other words, an animal that consumed 1 calorie of Ensure in rewards during the session received 1 less calorie of dry food in its diet. We also determined whether the animals were lethargic or showed any signs of dehydration. If their body weight dropped to <80% of their normal body weight, we then supplemented their diet with additional dry food on a daily basis. We progressively increased the daily amount of dry food that the mouse received, in 0.25 g increments, until the mouse reached the target 80% body weight.

### Training schedule

The training schedule consisted of six phases (designated phase 0 through phase 5). We did not set a fixed duration limit to each phase, as we found that by allowing some flexibility in the time required to reach benchmark criteria, we were able to train ∼90% of mice to successfully discriminate flickering stimulation. The total duration of the training period varied from 3 to 4 weeks, depending on the mouse. Any single mouse ran only one session per day, on consecutive days. Depending on the training phase, an experimental session consisted of anywhere from 300 to 500 trials, where a trial is defined as the period between the time when a “cueing” tone is turned on (start of trial) and the time when the tone is turned off (end of trial), regardless of whether the mouse engages in the expected task behavior (Fig. 1). Trial duration was set at 6–12 s, depending on the training phase (see below). All training was performed using green, 505 nm LED light stimulation presented at either 32 or 309 scotopic illuminance units (s lux). Flicker modulation was 12 Hz at 60% or 70% contrast, while nonflicker stimulation was same as the background luminance.

#### Phase 0

Mice learn to associate the presentation of a “heads-up” high-frequency (brief) tone with a visit to the reward tray. This is the sequence of events that mice will eventually follow to initiate a trial. Mice were allowed 12 s between the presentation of the heads-up tone and their visit to the reward tray, or the trial was aborted. Immediately after head entry into the reward tray, a low-frequency cueing tone was turned on for 6 s. A small amount of Ensure reward (∼5 μl) was delivered in the reward tray for reinforcement. Up to 400 trials were run per session.

#### Phase 1

Mice learn to pair head entry in the reward tray with a nose poke at a response port. During this training phase, the right nose-poke port is open while the left nose-poke port is covered with tape. The steady light stimulus paired with the cueing tone is presented after the mouse initiates the trial. Poking the open response port will result in reinforcement. To complete phase 1 mice will have to perform >300 trials per session for 3 consecutive days.

#### Phase 2

In this phase. mice are trained to associate a flickering stimulus paired with the cueing tone with a visit to left nose poke. During this training phase, the left nose-poke port is open while the right nose-poke port is covered with tape. The setup is similar to that described above for phase 1. To complete phase 2, mice will have to complete >300 trials/session for 3 consecutive days.

#### Phase 3

Mice are trained to associate flicker with the left nose-poke port and nonflicker stimulus with the right nose-poke port when the two nose-poke ports are open. The stimulus paradigm consists of consecutive blocks of three flicker presentations followed by three nonflicker presentations. Mice receive reinforcement only when they correctly visited the left nose-poke port during a flickering stimulus (Hit) or the right nose-poke port during a nonflicker stimulus (i.e., CR). Mice receive continuous reinforcement for correct responses. As in phase 2, mice have to complete >300 trials/session and achieve session performance (correct attempts/total attempts) >70% for 3 consecutive days, where correct attempts = Hits + CR and total attempts = Hits + CR + FA + Miss.

#### Phase 4

Mice learn to perform the visual discrimination task using a corrective procedure. The two nose-poke ports are open, and mice are presented in random order with either flickering or nonflickering light stimuli. Mice initiate the trials by visiting the reward tray following the heads-up tone. Trial durations are 6 s in duration. We compute correct responses when mice visit the left response port if flickering light is delivered (Hit) and visit the right port if a nonflickering light is delivered (correct rejection). Mice receive continuous reinforcement for correct responses. Incorrect responses are followed by up to four corrective trials. A corrective trial repeats the same stimulus that caused the incorrect response. Up to 500 trials are performed per session, and mice have to achieve a session performance >80% for 3 consecutive days. We refer to this corrective procedure as “cycling.”

#### Phase 5

Mice perform the discrimination task without the corrective procedure. Here we introduce *d*´ as a measure of performance. In general, the value of *d*´ first increases gradually in successive sessions as the mice learn the procedure, and eventually converges to a steady value. In the case of C57BL/6J mice, under these training stimulus conditions (505 nm LED lights, 32 s lux steady illumination, and flickering stimulus at 12 Hz, and 60% contrast), *d*´ approaches values that range from 1.5 to 2. We consider that mice have completed their training when *d*´ values remain within this range for 3 consecutive days. Note that every session in phase 5 (and later during “true” experiments) begins with a warm-up period, in which we run the cycling routine for 300 trials. In general, the warm-up period lasts 45–60 min.

#### Phase 6

Mice are trained to respond to four to six different contrast levels during a single session. This paradigm is used to measure psychometric functions (PFs). The training period is ∼3–4 d, which is sufficient time to build stable, linear psychometric functions with *R* ^2^ > 0.7.

#### Dealing with one-sided behavior during experimental sessions

On occasion, mice will stop visiting one of the two nose-poke ports, limiting their attention to either the right or left nose-poke ports. This lopsided behavior is occasionally observed in the first month after training has been completed but is also observed in more experienced mice performing challenging discrimination tasks (e.g., detection of flicker at high temporal frequencies or low contrasts that yield *d*´ < 1.0). As a rule, we abort the sessions where lopsided behavior is observed and run the corrective cycling routine described above. This strategy remedies the lopsided behavior in one or two sessions.

### Determination of retinal irradiance in freely behaving mice

Retinal irradiance depends both on the level of illumination at the cornea and the area of the pupil controlling the amount of light reaching the retina. Corneal irradiance (in watts/area) was measured with an M370 Optometer (Graseby Optronics) and converted into photopic (p lux) and scotopic (s lux) illuminance units using established formulas (Wyszecki and Stiles, 2000). Pupil areas of mice were measured as a function of corneal illuminance. To better replicate experimental conditions, we measured the pupil areas of unrestrained, behaving mice, using a custom-built portable device that automatically acquires close-up images of mouse eyes inside the operant behavior chamber, as described by Bushnell et al. (2016). Using this information, we applied the equations derived by Lyubarsky et al. (2004) to determine the levels of retinal irradiance at each experimental condition (Table 1). Note that we express retinal irradiance in terms of photon flux at the retina (ph/s/μm^2^) and not in the conventional form (R^*^/rod/s, where R^*^ are photoisomerizations) because rod effective collecting area values are likely to change following prolonged bleaching (2–3 h) at the higher irradiance levels used in the behavioral experiments (Lyubarsky et al., 2004).

**Table 1.** Corneal illumination, pupil size, and corresponding retinal irradiance

Corneal irradianceat 500 nm (ph/s/μm^2^)	Photopic corneal illumination (p lux)	Scotopic corneal illumination (s lux)	Pupil area (mm^2^)		Retinal irradianceat 500 nm (ph/s/μm^2^)		Rate of isomerizations per rod (R*/rod/s)	
			WT	G2	WT	G2	WT	G2
460,000	47.4	309	0.33	0.43	8200	11000	7200	9600
48,000	4.9	32.2	1.12	1.64	3000	4400	2600	3800
5000	0.51	3.36	1.58	1.48	440	410	380	360
54	0.0055	0.0363	3.09	3.85	9.2	1.1	8	10
Dark	Dark	Dark	3.72	3.83	NA	NA	NA	NA

### Quantification and statistical analysis

For the temporal contrast sensitivity functions, a two-way repeated-measures (RM) ANOVA was used with the nominal factors being irradiance and temporal frequency. The Holm–Sidak procedure for pairwise multiple comparisons was performed to test the hypothesis that mean measurements (TCS or slope of psychometric functions) did not differ with irradiance or frequency. When necessary, logarithmic transformations of contrast sensitivity data were performed before statistical analysis to fulfill normality and equal variance requirements for the ANOVA. Data analysis was performed with SigmaStat software (Systat Software). Plots display the mean ± SEM. The numbers of mice and *p* values are indicated in the figure legends.

## Results

### A forced-choice operant assay to measure TCS in mouse

We applied an operant behavior assay to determine TCS in mice using a yes–no (one-forced choice) paradigm (see Materials and Methods). Following training (for details, see Materials and Methods), we tested daily the ability of mice to distinguish flickering from nonflickering overhead lights (Fig. 1*A*). During the sessions, mice were continuously exposed to a fixed adapting light. The flicker test consisted of a sinusoidally varying full-field illumination superimposed on the steady adapting light (Fig. 1*B*). The level of the nonflicker test light was matched to the adapting light. Stimulus variables defined by the experimenter were mean light intensity, temporal frequency, and contrast. Presentation of flicker versus nonflicker stimuli in a trial was chosen randomly (probability of flicker = probability of nonflicker = 0.5). Output variables compiled at the end of each session were number of Hits (correctly reporting presence of flicker), false alarms (report flicker when nonflicker presented), misses (report nonflicker when flicker presented), and correct rejections (correctly reporting presence of nonflicker; Fig. 1*C*, Movie 1).

Movie 1. Short clip showing the behavioral responses of a mouse during a typical test session. The flicker test consisted of a sinusoidally varying full-field illumination superimposed on the steady adapting light. Stimulus variables were mean illuminance of 309 s lux delivering an estimated 8200 ph/s/μm^2^ at the retina, temporal frequency of 18 Hz, and 70% contrast. Presentation of flicker vs nonflicker stimuli in a trial was chosen randomly and with equal probabilities of 0.5. The mouse was trained to visit the left nose-poke port (near the bottom of the image) when it detects flicker, and the right nose-poke port in response to nonflicker stimulus. Output variables were the number of Hits (correctly reporting presence of flicker), FAs (report flicker when nonflicker presented), Misses (report nonflicker when flicker presented), and CRs (correctly reporting presence of nonflicker), as indicated by score table on the top of the video. See main text for details. The mouse was rewarded after each correct response (Hits and CRs) with a small amount of Ensure pumped into the reward tray. The activation of the pump during delivery can be heard in the audio track. Download Table 2-1, PDF file. 10.1523/ENEURO.0181-18.2018.video.1

### Application of the theory of signal detection to separate bias from sensitivity

Because the percentage correct metric is prone to response bias, we applied the TSD to estimate *d*´ (Eq. 1), a measure of performance that is independent of response bias and motivation. This approach is frequently used in psychophysical studies of sensory systems (Green and Swets, 1988; Macmillan and Creelman, 2005). TSD separates bias from sensitivity by assuming normality and homoscedasticity of the stimulus distributions; *d*′ summarizes discriminability as the separation of the distribution means in units of their common SD. In the mouse, the pairs of hit versus false alarm rates measured at different days clustered along an ROC curve, as predicted by TSD (Fig. 1*D*,*E*). The best fitting ROC curve was dependent on the value of contrast in the flickering stimulus, shifting toward the top left corner of the plot as contrast values increased (Fig. 1*D*,*E*).

### Values of *d*´ derived from hit versus FA pairs were stable during daily sessions

**Figure 2.**
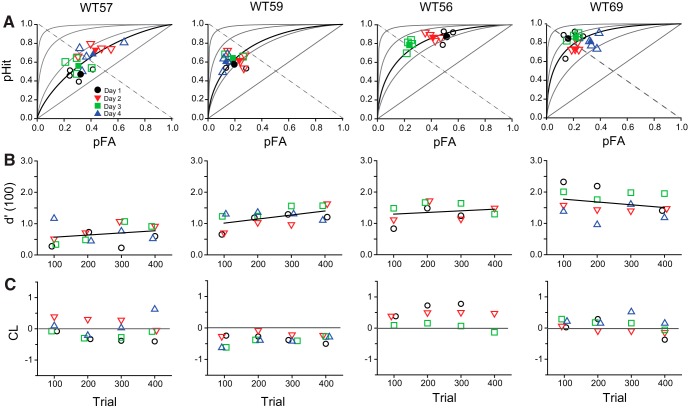
Values of *d*´ were stable during daily sessions. *A*, Hit vs FA pairs for four WT mice measured on different days clustered about the ROC curves implied by the pooled value of *d*´. Stimulus conditions (flicker frequency, contrast, illuminance at the cornea, and retinal illumination) for WT56, WT57, and WT59 were as follows: 12 Hz, 50%, 3000 ph/s/μm^2^; for WT69, 21 Hz, 40%, 8200 ph/s/μm^2^. The values of *d*´ for each mouse were: WT57, 0.65; WT59, 1.18; WT56, 1.32; and WT69, 1.63, respectively. For comparison, ROC curves for *d*´ = 0 (main diagonal), 1, 2, and 3 are also shown (gray ROC curves, rising in order away from the main diagonal). The negative diagonal (dashed line) indicates unbiased behavior. Open symbols, Hit vs FA pairs every 100 trials; closed symbols, daily totals (400 trials). *B*, Values of *d*´ for the corresponding 100 trial blocks (estimated with Eq. 1; Materials and Methods). *C*, Changes in response bias expressed in terms of criterion location (Eq. 2; Materials and Methods). Note that for clarity purposes some of the symbols have been displaced laterally.

Next, we investigated for potential intrasession trends in performance (or habituation) during the 400 trials in 2- to 3-h-long sessions. We tested four mice over the course of 3–4 d and divided each session into four sequential bins, each with 100 trials/bin. Hit versus FA pairs for four WT mice measured on different days clustered about the ROC curves implied by the pooled value of *d*´ (Fig. 2*A*). To test the stability of the measurements, we determined the values of *d*´ for each bin and plotted them as a function of bin number (Fig. 2*B*). The plots show variability in *d*´ values in within-day (intrasession) as well as in day-to-day comparisons. However, linear regression analysis indicates no systematic intrasession trend in *d*´ values (slope not significantly different from 0, *p* > 0.31) for WT57, WT56, and WT69, while a slow increase in *d*´ values was observed for WT59 (slope = 0.0013 *d*´ units/100 trials; *p* = 0.031). These small or statistically insignificant intrasession trends in the value of *d*´ rule out habituation or other systematic behavioral trends during our experimental sessions. Day-to-day variation is examined in detail below.

### Behavioral bias changes significantly during the measurements of hit versus FA pairs

Behavioral bias can be inferred qualitatively from the ROC curves in Figure 2*A.* Hit versus FA pairs for WT57 and WT69 cluster about the negative diagonal of the plot (dashed gray line), which is consistent with unbiased behavior. In contrast, Hit versus FA pairs for WT59 clustered below the diagonal, which is indicative of biased behavior that favors the right over the left nose-poke port, while the pairs for WT56 are largely above the diagonal, which is consistent with bias for the left nose-poke port. Response bias was quantitatively expressed in terms of CL (Materials and Methods; Eq. 2). A value of CL = 0 corresponds to unbiased behavior, while CL > 0 indicates bias favoring the left port (FA rate > Miss rate), and CL < 0 indicates bias favoring the right port (FA rate < Miss rate). Our data show a relatively unbiased response behavior for WT69; however, WT59 exhibited a consistent negative bias while WT56 exhibited a positive bias (Fig. 2*C*). WT57 oscillated back and forth between negative and positive biases.

### No significant long-term trends in *d*´

**Figure 3.**
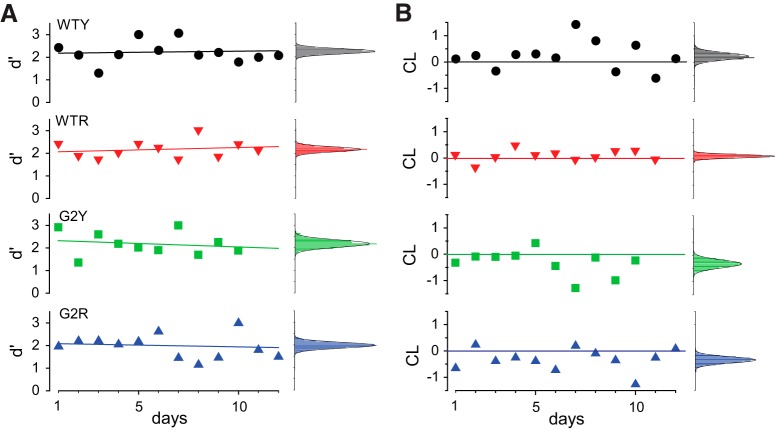
No significant long-term trends in *d*´. *A*, *d*´ values varied from day to day about a steady mean. *d*´ values of two C57BL/6J mice (WT) and two mice GNAT2*^*cpfl3*^* (G2) over the course of 10–12 consecutive days. The daily experimental sessions consisted of 100 trials in response to sinusoidal flicker stimulation. Individual contrast levels were adjusted during a pretrial run to elicit a *d*´ = 2 and were kept constant thereafter. Continuous lines represent linear regression fits (see text). The histograms at the right of each plot show the distribution of average *d*´ values estimated by sampling with replacement (*n* = 400 bootstrap samples) of the data in the respective plots. Flicker parameters were 21 Hz presented on a background illuminance level producing an estimated retinal irradiance of 8200 ph/s/μm^2^. *B*, Corresponding values of CL. Continuous lines represent the zero (unbiased behavior) axis.

Next, we examined day-to-day variation and bias. We tracked *d*´ and CL in two C57BL/6J (WT) and two GNAT2^*cpfl3*^ (G2) mice over a period of 10–12 d. The daily experimental sessions consisted of 100 trials in response to sinusoidal flicker stimulation with the same contrast. Values of *d*´ values varied from day-to-day about a steady mean (Fig. 3*A*). The slightly increasing or decreasing trends in *d*´ were not statistically significant (linear regression analysis, *p* > 0.6). The histograms at the right of each plot in Figure 3*A* show the distribution of average *d*´ values estimated by sampling with replacement (*n* = 400 bootstrap samples). Day-to-day values of *d*´ were normally distributed (Shapiro test, *p* = 0.323) with equal variance across mice (*p* = 0.115). For each mouse, the mean and SD values for *d*´ were as follows: WTY, 1.98, 0.32; WTR, 1.99, 0.35; G2Y, 1.9, 0.25; and G2R, 1.86, 0.24, respectively.

CL plots illustrate the daily changes in bias throughout the test period (Fig. 3*B*). G2R consistently favored the right nose-poke port (CL < 0), while WTY and G2Y exhibited biased behavior during the second half of the testing period. The histograms at the right of each plot show the distribution of average CL values estimated using bootstrapping with replacement (*n* = 400). Mean and SD values for CL were as follows: WTY, 0.074, 0.19; WTR, 0.15, 0.17; G2Y, −0.17, 0.25; and G2R, −0.2, 0.34. Likewise, CL was normally distributed (*p* = 0.623) and equal in variance across mice (*p* = 0.31). These results indicate reproducible day-to-day performance, as measured by *d*´.

### *d*´ is a bias-free measure of temporal contrast sensitivity in mice

**Figure 4.**
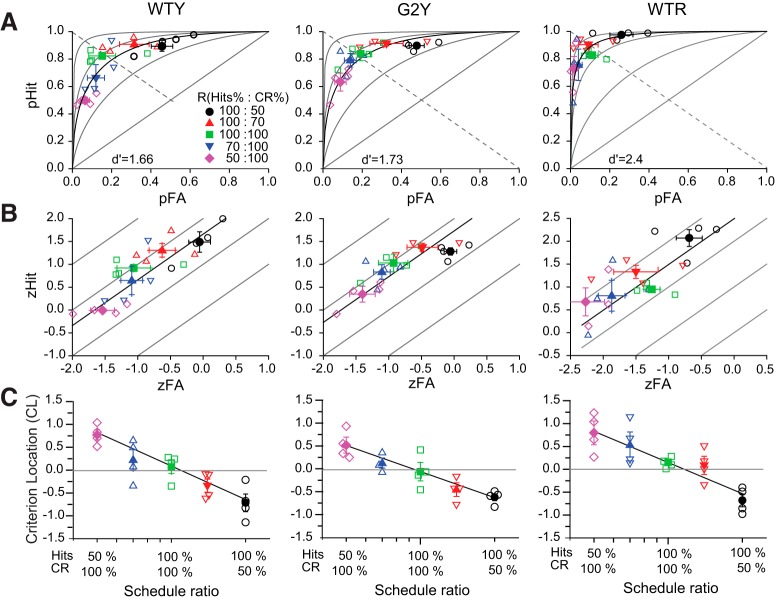
*d*´ is a bias-free measure of temporal contrast sensitivity in mice. *A*, ROCs implied by *d*´ can predict empirical data. Empirical ROC curves of three mice (two WT mice and one GNAT2*^*cpfl3*^* mouse labeled WTY, WTR, and G2Y, respectively) determined by varying the reward schedules. Mice were first tested with 100%:100% reward schedules, and successively with 100%:70% and 100%:50% reward schedules, or alternatively, with 70%:100% and 50%:100% reward schedules. Open symbols indicate daily pHit and pFA pair values, closed symbols represent the average ± SEM, *n* = 5-6 trials. The data are well fit by the model: *d*´ = *z*(pHit) − *z*(pFA), where *z*(pHit) and *z*(pFA) are the *z* score values (in SD units) of pHits and pFA. Model predictions are illustrated by the continuous ROCs with maximum likelihood predicted *d*´ values of 1.66 (WTY), 2.49 (WTR), and 1.73 (G2Y; Table 2). For reference, ROCs for *d*´ = 0, 1, 2, and 3 are indicated in gray. *B,* Same as in *A*, but plotted on *z*-coordinates. Note that in these coordinates the ROCs have unitary slopes, which is consistent with the notion that *d*´ sensitivity estimates do not depend on the location of the decision criteria (Macmillan and Creelman, 2005). *C*, CL derived from the empirical ROC curves decreases linearly with the logarithm of the reward schedule ratio. Continuous black lines indicate regression fits (*R* ^2^ > 0.9) with Equation 5, and *q* values of 1.75 (WTY), 2.4 (WTR), and 1.4 (G2Y).

Our behavioral results (Figs. 2, 3) indicate that behavioral bias changes significantly during measurements of Hit versus FA pairs. In the context of TSD, changes in bias reflect variations in dynamic decision criteria that can result in inaccurate estimations of *d*´ unless the shape of the ROC curve is known and taken into account during the analysis of the data (Macmillan and Creelman, 2005). ROC curves can be determined empirically, by rating the degree of confidence that subjects have in making their decision (McNicol, 1972). Because these rating approaches are generally not applicable to mouse behavioral studies, we generated their empirical ROC curves by extrinsically manipulating response bias via manipulation of the reward schedule (Fig. 4). An alternative approach—manipulation of flicker versus nonflicker stimulus presentation probabilities—did not systematically alter mouse behavior in our hands and was not pursued further.

We manipulated the reward schedule while holding the probability of stimulus presentation constant and balanced (50% flicker vs 50% nonflicker). Mice were tested in daily experimental sessions where they were first exposed to 300 trials on a 100%:100% reward schedule to determine their baseline bias, and then followed, in succession, by 300 trials on 100%:70% and 300 trials on 100%:50% reward schedules (note that the numerator and denominator in the ratio refer to the percentages of Hits and CRs rewarded, respectively). To allow for the transition to the new “bias” state after switching reward schedules, Hits versus FA pairs were evaluated only during the last 100 trials of each 300 trial block. Given the variability observed in *d*´ and CL values (Figs. 2, 3), the procedure was repeated four to six times on separate days, and the corresponding *d*´ and CL values for each reward schedule were averaged. A similar procedure was applied to estimate *d*´ and CL values in response to 70%:100% and 50%:100% reward schedules.

For this study, we selected two wild-type mice (WTY, WTR) and one GNAT2*^*cpfl3*^* mouse (G2Y) that exhibited unbiased behavior in response to 100%:100% reward schedules (one other mouse in the group did not respond to extreme ratio schedules and was therefore not included in the analysis). Contrast levels were adjusted individually during a preliminary session so that mice performed with a *d*´ value equal to ∼2 and then kept constant in all the subsequent testing sessions. The unbiased behavior was confirmed by the distribution of the pHit versus pFA pairs (green symbols) along the negative diagonal (dashed line) of the plots (Fig. 4*A*), where pHits and pFA indicate the probability of Hits and FA respectively. When tested with 100%:70% and 100%:50% reward schedules, the corresponding responses exhibited a progressive increase in both pHit and pFA (as mice favored the left over the right nose-poke ports) and the location of the pHit versus pFA pairs shifted toward the top right corner of the plot. Conversely, when tested with 70%:100% and 50%:100% reward schedules, the pHit and pFA response rates decreased (as mice now favored the right over the left nose-poke port), prompting a shift of the pHit versus pFA pairs toward the bottom left corner of the plot. When plotted on *z*-coordinates, the data points cluster about ROC curves with unitary slopes (Fig. 4*B*), which is consistent with the notion that *d*´ sensitivity estimates do not depend on the decision criteria (Macmillan and Creelman, 2005).

To validate the application of TSD to our mouse operant behavior, we determined whether the ROC curves implied by *d*´ can predict the empirical data. We used maximum-likelihood estimation (PALAMEDES toolbox; Prins and Kingdom, 2018) to compute the best fitting values for *d*´, the standard deviation (SD) of the signal (flicker)-to-noise (nonflicker) ratio, and their corresponding standard errors (SEs). PALMEDES uses the Nelder–Mead Simplex method to maximize the likelihood that the observed data were produced by the proposed ROC curve and parametric bootstrapping to determine the SE of the best fitting parameters (800 simulations). The results (summarized in Table 2) indicate that the predicted ROC curves are compatible with a model in which the underlying distributions characterizing the response probabilities to flicker (the signal) and nonflicker (noise) have the same SD value (ratio-of-SD-different-from-1 *p* value > 0.05) and confirm that *d*´ is defined by Equation 1 (goodness-of-fit *p* values > 0.05).

**Table 2.** Best fitting parameters for the estimated ROC curves of three different mice shown in Figure 4A–C

Parameter	WTY	G2Y	WTR
*d*´	1.66	1.73	2.49
*d*´ SE	0.066	0.07	0.08
SD ratio	1.03	0.79	1.02
SD/SE ratio	0.15	0.15	0.24
Goodness-of-fit *p* value	0.06	0.08	0.1
Ratio SD different from 1 *p* value	0.84	0.14	0.91
Convergence ratio	800/800	800/800	800/800

*d*´SE = SE of *d*´; ratio SD = ratio of signal (flicker) to noise (nonflicker) SDs; convergence ratio = number of simulations that converged to total number of simulations.

Next, we examined how the reward schedule alters behavioral bias. Visual inspection of the data suggests that the CL is linearly related to the logarithm of the reward schedule ratio (Fig. 4*C*). This relation is well fit by the following expression (see derivation in Materials and Methods):

(5) $$\text{CL}=\text{CL}\left(100\text{\%},100\text{\%}\right)-\frac{q}{d^{\prime }}\text{ln}\left[\frac{R\left(\text{Hit}\right)}{R\left(\text{CR}\right)}\right],$$

where *R*(Hits)*/R*(CR) is the schedule ratio and the constant term CL(100%,100%) accounts for the intrinsic bias exhibited by each mouse when tested with a balanced reward schedule [*R*(Hit) = *R*(CR) = 100%]. The values of CL(100%,100%) and *d*´ were determined experimentally, while the schedule ratio *R*(Hit)/*R*(CR) is the independent variable. The ratio *q*/*d*´ determines the slope of the function, where *q* is the only free variable. Regression analysis shows that this simple model fits the empirical criterion locations for the three individual mice reasonably well (*R* ^2^ > 0.9) with *q* values of 1.75, 1.4, and 2.4 for WTY, G2Y, and WTR, respectively. In all, these results show the following: (1) mouse decision criteria can be manipulated by varying the reward schedule ratios; (2) the shape of empirical ROC curves is predicted by TSD, and (3) *d*´ is a bias-free measure of flicker sensitivity in mouse.

### Psychometric functions are linear and have constant slopes

**Figure 5.**
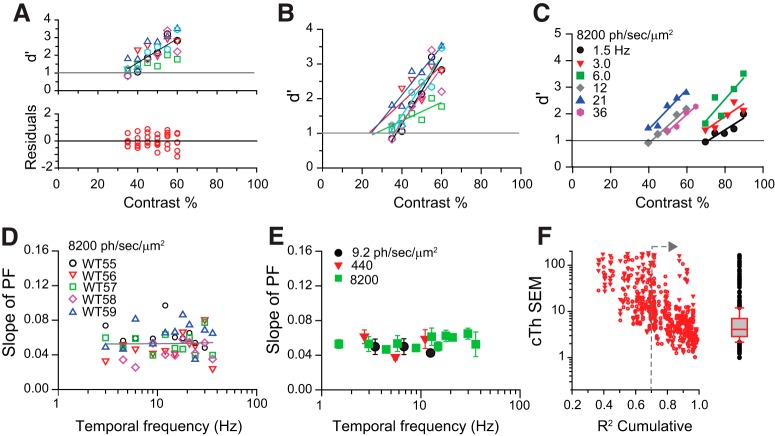
Linear psychometric functions and determination of empirical thresholds. *A*, Top, Representative set of PFs for a WT mouse measured on different days. Flicker stimulus parameters were 18 Hz flicker and a mean irradiance level of 3000 ph/s/μm^2^. The contrast range sampled was 35–60% in 5% intervals. Linear regression parameters were cTH = 31%, slope = 0.068 *d*´/% contrast, *R* ^2^ = 0.58, *p* < 0.001. Bottom, Scatter plot of the residuals. Consistent with linearity. we did not observe any bends, thinning, or thickening in the scatter of the residuals. *B*, Linear fits of the PFs in *A*. The respective cTh values were determined at the point where the extrapolated lines intersect the line defined by *d*´ = 1. In this dataset, threshold values ranged from 20% to 35% contrast. *C*, Family of PFs measured from a WT mouse in response to flickering lights at the indicated frequencies. The mean retinal irradiance was 8200 ph/s/μm^2^. Each PF represents the cumulative average of two to three measurements. Linear regression (*R* ^2^ > 0.70) was applied to determine the slope and empirical threshold where each PF intersects the line defined by *d*´ = 1. *D*, Slopes of PFs do not change with flicker frequency. The plot shows slopes of PFs vs frequency for five different mice at mean background light levels delivering 8200 ph/s/μm^2^. The slope of the PFs remains constant and does not change with frequency (linear regression, *p* = 0.8). *E*, Slopes of PFs are constant over the mesopic range. The plot compares the mean values of the PF slopes measured at 8200 (black circles), 440 (red triangles), and 9.2 (green squares) ph/s/μm^2^. Note that mice do not respond to flicker frequencies >24 Hz when exposed to 440 ph/s/μm^2^, or >12 Hz when exposed to 9.4 ph/s/μm^2^. Pairwise comparisons at 3, 6, and 12 Hz reveal no significant differences in mean slope values (two-way RM ANOVA, Holm–Sidak method, α = 0.05). Symbols represent the average ± SEM (*n* = 5). Note that for clarity purposes some of the symbols have been displaced laterally. *F*, cTh_SEM values estimated from cumulative averages of two (circles) or three (triangles) PFs using bootstrapping techniques and plotted as a function of cumulative *R* ^2^. Box plot indicates distribution quartiles for cumulative *R* ^2^ > 0.70 (indicated with gray arrow in plot).

To determine temporal contrast sensitivity in mice, we first measured their psychometric functions (PFs). PFs are plots of *d*´ values in response to different contrast levels (Fig. 5). The (empirical) contrast threshold, which depends on the relative shift of the PF along the contrast axis, was arbitrarily defined as the contrast necessary to elicit a *d*´ = 1, which by TSD produces 76% correct responses in alternative forced-choice tasks and is bias free. TCS is the inverse of the threshold.

Full PFs were determined in single daily sessions consisting of 400 trials, where the magnitude of the contrast applied at each trial was variable and selected randomly from an array of five to six possible contrast values (separated by 5% contrast intervals). Background illumination level and flicker frequency were the independent variables. PFs are relatively linear for contrast values eliciting *d*´ < 3 (Fig. 5*A*). Hence, we applied linear regression and determined the empirical contrast threshold at the intersection of the regression line with *d*´ = 1. To reduce the uncertainty associated with day-to-day variability (Fig. 5*B*), we measured and averaged PFs repeatedly until the regression coefficient *R* ^2^ for the cumulative average was ≥0.70, a process that requires averaging two to three PFs.

A representative family of PFs for a WT mouse is shown in Figure 5*C*. The position of the PFs along the contrast axis depends on the frequency of the flicker stimulus. However, the slope of the PFs does not change with frequency (Fig. 5*D*) or background illumination levels (range, 9.2–8200 ph/s/μm^2^; Fig. 5*E*). These results suggest that the sensitivity to systematic changes in contrast is independent of flicker frequency and background illumination level tested.

We used bootstrapping techniques to assess the standard error (SE) associated with the contrast threshold measurements (Efron and Tibshirani, 1994). We first generated four different sets of PFs from WT mice, each set consisting of four to seven PFs (Fig. 5*B*). Next, we randomly selected and averaged two (or three) PFs in a given set, ran a linear regression on the average, and applied nonparametric bootstrap on the residues (*n* = 1000 samples) to determine the SE of the thresholds (cTh_SEM) and corresponding *R* ^2^ values. Next, a new pair (or triplet) of PFs from the same set was randomly selected, and the procedure was repeated to generate a new estimate of cTh_SEM. This process was repeated 100 times to estimate the distribution of cTh_SEM values for each set of PFs.

Our analysis indicates that cTh_SEM decreases gradually as the cumulative *R* ^2^ increases (Fig. 5*F*). For cumulative *R* ^2^ > 0.7 (as per our experimental conditions), the cTH_SEM has an estimated median value of 5% contrast (25% quartile = 3.0% contrast, 75% quartile = 8.0% contrast). No significant differences were observed in the distributions of cTh for pair and triple cumulative averages (Fig. 5*F*, circles vs triangles, respectively). Taking a conservative view, we can expect that cTh_SEM for each mouse will be on the order of 8–10% contrast and down to ∼5% contrast after averaging results in groups of four to six mice. Such a degree of variability agrees with our practical observations (see below).

### Mouse TCS functions have classic properties of human TCSF

**Figure 6.**
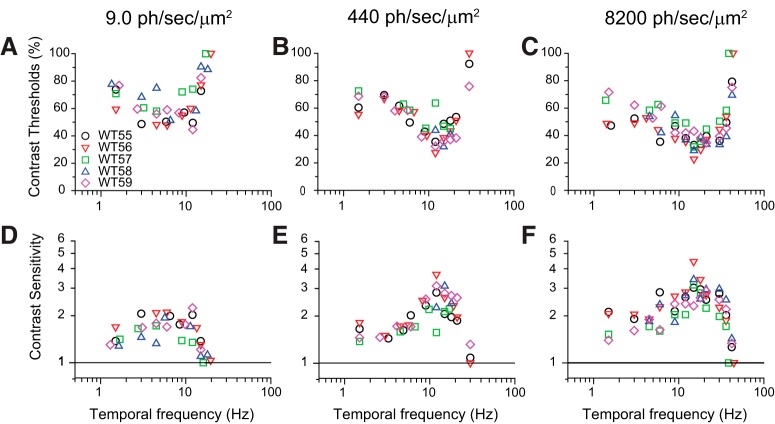
Contrast thresholds decrease with mean irradiance. *A–C*, Empirical thresholds of WT mice measured as a function of flicker frequency determined at three different background illumination levels, dim (9.2 ph/s/μm^2^ at the retina; ***A***), intermediate (440 ph/s/μm^2^; ***B***), and bright (8200 ph/s/μm^2^; ***C***). The contrast thresholds for each mouse were determined from families of psychometric functions, as defined in Figure 5*C*. Note that for clarity purposes some of the symbols have been displaced laterally. ***D–F***, Corresponding TCSFs estimated from the contrast threshold functions in ***A–C***.

To determine TCS functions, we determined the empirical contrast thresholds (as described above) at multiple temporal frequencies and irradiance levels (Fig. 6*A–C*). The irradiance levels spanned almost two orders of magnitude from 9.2 to 8200 ph/s/μm^2^, while the temporal frequencies ranged from 1.5 Hz to the highest frequency that elicited a behavioral response, that is, between 20 and 40 Hz, depending on irradiance levels. The plots show that the contrast thresholds decreased while the bandwidth increased with increasing irradiance levels. The SEM in all these measurements was generally <5% contrast, which is consistent with the error estimations detailed above. Next, we determined the corresponding TCS as the inverse of the contrast thresholds and plotted the TCSFs at each background level (Fig. 6*D–F*).

**Figure 7.**
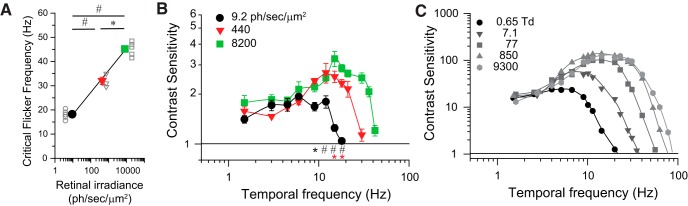
Mouse TCSFs have classic properties of human TCSFs. *A*, CFF increases with irradiance. Statistical analysis: RM ANOVA, significant differences between groups, #*p* < 0.001, *p < 0.05, with Bonferroni method. Symbols represent the average ± SEM (*n* = 3–5; for values, see Fig. 7-1). Linear fit represents predictions according to the Ferry–Porter law (see text for details). *B*, Comparison of average mouse TCSFs in Figure 6*D–F* at indicated irradiance levels. Statistical analysis: two-way RM ANOVA, #*p* < 0.001; *p < 0.05, comparisons for factor irradiance at 8200 vs 9.2 and 8200 vs 440 ph/s/μm_2_ are indicated with black and red symbols, respectively, using the Holm–Sidak method (see text for details). Symbols represent the average ± SEM (*n* = 5; for values, see Fig. 7-1). *C*, Human temporal contrast sensitivity functions adapted from Kelly (1961) illustrate the classic properties of adaptation to increasing illuminance levels (see text). Retinal illuminance levels for each function are indicated in Trolands (Td) and can be converted to equivalent rates of photoisomerization by applying the conversion relation previously established for primates wherein 1 Td = 11 R*/rod/s (Hood et al., 1999).

In dim backgrounds eliciting 9.2 ph/s/μm^2^ in the retina, the TCSF had a characteristic low-pass shape. TCS was approximately constant for frequencies up to 12 Hz, with a value of 1.7–1.9, and fell sharply for frequencies >12 Hz (Fig. 6*D*). The Critical Flicker Frequency (CFF), defined as the frequency where the TCSF intersects the line defined by TCS = 1, was equal to ∼18 Hz. CFF is the highest temporal frequency that a mouse can detect at a flicker contrast of 100% and provides a measure of temporal resolution. CFF was determined by extrapolation of the last two TCS values in the sharp, high-frequency asymptote of individual TCSFs. As irradiance levels rose to 440 ph/s/μm^2^, the TCSF acquired a bandpass shape, with a peak sensitivity of 2.6 at 12 Hz (Fig. 6*E*). TCS fell gradually with low frequencies, reaching a steady level of 1.5–1.7 in response to frequencies <6 Hz, while it fell sharply in response to high frequencies, resulting in a CFF of 30 Hz. At 8200 ph/s/μm^2^, the TCSF maintained the bandpass shape, but with a wider bandwidth and a CFF of 42 Hz (Fig. 6*F*). The increase in CFF with retinal irradiance (Fig. 7*A* and Fig. 7-1) was statistically significant (*p* < 0.001; individual comparisons indicated in plot; RM ANOVA, Bonferroni *t* test method). Moreover, CFF increases in proportion to the logarithm of irradiance, and the relation is well fit (*R*^2^ > 0.99) by the following equation:

(6) $$\text{CFF}=a+K\text{ log }(I_{B}),$$

where *a* = 9.0 Hz is the vertical offset constant, *K* = 9.1 Hz/decade is the slope of the relation, and *I_B_* is the retinal irradiance. These results are in agreement with the Ferry–Porter law and indicate a general speeding up of the visual processes with light adaptation (Tyler and Hamer, 1990).

10.1523/ENEURO.0181-18.2018.f7-1 Figure 7-1 Extended data legend. Download Figure 7-1, PDF file.

10.1523/ENEURO.0181-18.2018.t1-1 Table 1-1 Temporal contrast sensitivity vs temporal frequency. Download Table 1-1, PDF file.

10.1523/ENEURO.0181-18.2018.t2-1 Table 2-1 Critical flicker frequency vs retinal irradiance. Download Table 2-1, PDF file.

A number of important properties of temporal vision emerge when we compare TCSF measured at various levels of illumination (Fig. 7*B* and Fig. 7-1). Indeed, our results show that TCSFs in mice share many key features with TCSFs in humans (compare Fig. 7*C*, adapted from Kelly, 1961; for review, see Shapley and Enroth-Cugell, 1984; Watson, 1986), as follows: (1) Weber adaptation, in which contrast threshold remains constant in response to low temporal frequencies and increasing background illumination (no significant difference with frequencies <9.0 Hz, irradiance × frequency interactions were observed for frequencies >9 Hz (*p* < 0.001, power = 1), significance levels indicated in the plot; two-way RM ANOVA, Holm–Sidak method]; (2) a shift in the shape of TCSFs from low-pass to bandpass with increasing background illumination; (3) increased TCS with increased background illumination; and (4) increased temporal resolution (up to ∼40 Hz) with increasing background illumination (Fig. 7*A*), which is in line with the Ferry–Porter law (see above). Together, these data demonstrate that the mouse is an appropriate model of temporal contrast vision that shares fundamental properties of human psychophysics.

## Discussion

Understanding the neural processes that underlie the detection of temporal contrast in the mouse requires that we pair electrophysiological findings with rigorous psychometric measurements. Here we have developed an operant behavior assay that uses a forced-choice alternative-response protocol to determine TCS in mouse. We validated and applied TSD to estimate the *d*´ as an unbiased measure of sensitivity to flicker contrast. After measuring the psychometric functions, defined as plots of *d*´ versus flicker contrast, we determined the corresponding empirical thresholds at different illumination levels. Using this information, we generated the temporal contrast sensitivity functions and found that TCS in the mouse shares fundamental properties with human vision. Next, we discuss our major results.

A variety of go/no-go operant behavioral assays are frequently used to study the association between neural processes and behavior (Carandini and Churchland, 2013). However, these methods have a drawback in that they are subject to behavioral bias, which can increase the variability of the performance indexes being measured (Macmillan and Creelman, 2005). Here, we have shown, using a forced-choice approach, that bias in nose-poke port selection can be explained in terms of the theory of signal detection (Fig. 4) and a decision model that is based on the discrimination of flicker (signal) versus nonflicker (noise) distributions with the same SD values (Green and Swets, 1988). This important result not only highlights the advantage of using forced-choice rather than go/no-go protocols when probing mouse behavior, but also provides a working model for characterizing the activity of neurons along the visual pathway in response to temporal variations in light (Parker and Newsome, 1998; Smith and Dhingra, 2009). Although this method was designed to measure TCS in freely behaving mice receiving uniform retinal illumination (to be able to readily compute retinal irradiance levels), we believe that it can be readily modified to probe TCS and neural activity in head-fixed mice, as was used in the study by Burgess et al. (2017) in their forced-choice operant behavior assay.

Our second major finding is that mouse TCS functions have classic properties of human TCSF (Fig. 7). This is an important result that can help to guide the selection of experimental conditions required for electrophysiological studies, which, combined with the power of mouse genetics, can be designed to explore the neural basis of well established laws of temporal vision, as for example, Weber adaptation (Gesheider, 1997), the luminance-dependent increase in CFF (the Ferry–Porter law; Tyler and Hamer, 1990), or the transformation of the TCSFs from low-pass to bandpass shapes (de Lange, 1958; Kelly, 1961). The slope of the Ferry–Porter relation fitting the plots of CFF versus irradiance in mice (9.1 Hz/decade) is in close agreement with slope values reported previously for humans under photopic conditions (10–20 Hz/decade; Tyler and Hamer, 1990). CFF was ∼25 Hz at lower irradiance levels delivering 50 ph/s/μm2 (as interpolated from Fig. 7*A*) and was in line with the flicker fusion values reported previously by Nathan et al. (2006) using a water maze and temporal resolution, as inferred from the work of Peinado Allina et al. (2017) using a running wheel assay. We note, however, that although mouse and human share a number of fundamental properties in their temporal vision, they also differ in some aspects of overall performance. For example, at illumination levels delivering ∼8200 ph/s/μm^2^ at the retina, the CFF in the mouse was 42 Hz (Fig. 7), which is substantially higher than the highest temporal frequency that is typically measured with the optomotor response (15 Hz; Umino et al., 2008), but is lower than the CFF of humans when measured under the same illumination conditions [70 Hz (as inferred from Kelly, 1961)]. Interestingly, the CFF of 42 Hz is in line with the maximal frequency response recorded at the retina with photopic flicker ERGs (Krishna et al., 2002; Shirato et al., 2008) and at the dorsal lateral geniculate nucleus and V1 cortical areas of awake mice using multiunit recordings (Reinhold et al., 2015). A major difference between mice and humans relates to the absolute values of TCS. We find that typical TCS values in the C57BL6J mouse are on order of 2–3 (Fig. 7*B*), whereas TCS values in humans can reach 90–100 (Kelly, 1961; Watson, 1986). Such interspecies differences in visual properties are not exclusive to TCS. Differences have also been reported in the values of spatial contrast sensitivity in humans (100–300, Robson, 1966; van Nes et al., 1967) versus mice (5–10, Prusky and Douglas, 2004; Busse et al., 2011). However, one other study (Histed et al., 2012) suggests that spatial contrast sensitivity values in the mouse match those of human peripheral retina. In all, we have demonstrated that the mouse is a model of human vision that shares fundamental properties of human temporal psychophysics.

## Synthesis

Reviewing Editor: Leonard Maler, University of Ottawa Decisions are customarily a result of the Reviewing Editor and the peer reviewers coming together and discussing their recommendations until a consensus is reached. When revisions are invited, a fact-based synthesis statement explaining their decision and outlining what is needed to prepare a revision will be listed below.

The reviewers both found that this manuscript provides an excellent and sound analysis of temporal contrast sensitivity in the mouse. It is the first that separates bias from discriminability. This behavioral assay will be useful for not only studies of retinal function, but for higher level vision as well.

This Ms can therefore be accepted for publication with minor revisions described below. The revised Ms will only be reviewed by the Editor (Dr. Maler). I have provided the complete detailed reviews of both reviewers. Please make sure that your provide a detailed response to each point as well as a 'red-lined' version of the Ms. I will simply check to make sure that, wherever possible, you have complied with the very reasonable suggestions of the reviewers.

Both reviewers found that the video of the mouse performing the task was valuable and should be included.

Reviewer #1 Detailed comments

Page 12: The bright background that was used is clearly not scotopic, as suggested at several points in the manuscript. The conversion into human scoptopic illuminance units is probably not appropriate.

The figures are small and cramped, and symbols are not different enough from each other. Figures are very hard to read. These valuable plots of the data should be laid out more generously.

It would be useful for the authors to provide the exact values in their later figures in the extended data to facilitate their use by future workers, averting difficulties in scaling values from tiny figures.

The paper is missing citations to relevant literature on flicker responses in the mouse, including Reinhold...Scanziani 2015 Nature Neuroscience, Nathan...Hurley 2006 Photochemistry and Photobiology, and Jacobs...Fenwick 2004 Vision Research. The results from this paper should be contrasted with the results from these other papers in the Discussion.

Equation (4) has an additional negative sign that is not part of the derivation from (3). I believe this is an error, though the negations might be useful for equation (5). Please clarify.

Temporal contrast sensitivity (TCS) is not clearly quantified - does it simply equal d'?

Fig 6H must be mislabeled because the highest background illumination produces responses up to the highest temporal frequency.

It is surprising that the experiment was not counterbalanced for location such that the Flicker location should have been the right, and the Non-Flicker on the left for roughly half of the mice. This could prove important as it could rule out systematic bias in the motor behavior of the mouse. However, I do not think that this change should be required for publication.

Reviewer #2 Detailed comments

1. How does temporal contrast sensitivity (Fig. 6) change with frequency and background luminance for GNAT2 mice with reduced cone function? This would be essential to test if indeed such a behavioral assay can be used for relating retinal deficits to behavior.

2. It would be beneficial to the readers to simplify the text in the results and figures. The pHit Vs pFAplots could be bigger and it would help to have bar plots comparing different parameters like slope, etc.

3. In discussion it would help to mention the range of temporal sensitivity in the retinal output channels and compare that with cortical measures to give the readers an idea about possible mechanistic origin.

4. In Fig.2 it is not clear why the different mouse strains have such varied distributions.
