# Supplementary material for: Visual Temporal Contrast Sensitivity in the Behaving Mouse Shares Fundamental Properties with Human Psychophysics
Source: eNeuro. 2018 Aug 29;5(4):ENEURO.0181-18.2018. doi: 10.1523/ENEURO.0181-18.2018 (PMC6140104; doi:10.1523/ENEURO.0181-18.2018)
Supplement: Figure 7-1 — Extended data legend. Download Figure 7-1, PDF file. [file sup_enu-eN-NWR-0181-18-s02.pdf]

Extended data: Figure 7-1

	Retinal irradiance (ph/sec/μm^2 at 500 nm)					
	9.2		440		8200	
Frequency (Hz)	TCS	SEM	TCS	SEM	TCS	SEM
1.5	1.40	0.07	1.57	0.09	1.77	0.18
3.0	1.71	0.12	1.46	0.01	1.85	0.12
4.5	1.72	0.15	1.66	0.03	1.83	0.04
6.0	1.93	0.08	1.79	0.07	2.13	0.23
9.0	1.68	0.09	2.40	0.08	2.20	0.14
12.0	1.79	0.15	2.69	0.36	2.51	0.14
15.0	1.24	0.05	2.54	0.20	3.26	0.34
18.0	1.042	0.04	2.32	0.12	2.86	0.14
21.0			2.15	0.23	2.66	0.12
30.0			1.13	0.09	2.50	0.17
36.0					2.06	0.14
42.0					1.20	0.08

Fig. 7-1: Table 1. Average Temporal Contrast Sensitivity Functions (n = 5)

Retinal irradiance (ph/sec/mm2 at 500 nm)	CFF (Hz)	SEM
9.2	18.2	0.66
440	31.0	1.52
8200	45.2	1.15

Fig. 7-1: Table 2. Average Critical Flicker Frequency vs Retinal Irradiance (n = 3-5)
